# The Prognostic Value of Right Ventricular Function in Patients with Chronic Heart Failure—A Prospective Study

**DOI:** 10.3390/jcm13071930

**Published:** 2024-03-27

**Authors:** Nora Schwegel, David Zach, Alexander Peikert, Viktoria Santner, Viktoria Höller, Johannes Gollmer, Johannes Späth, Hermann Riepl, Peter P. Rainer, Markus Wallner, Stefan Pilz, Andreas Zirlik, Dirk von Lewinski, Klemens Ablasser, Nicolas Verheyen, Ewald Kolesnik

**Affiliations:** 1 Division of Cardiology, University Heart Center Graz, Medical University of Graz, 8036 Graz, Austria; 2 Department of Medicine, St. Johann in Tirol General Hospital, 6380 St. Johann in Tirol, Austria; 3 BioTechMed Graz, 8010 Graz, Austria; 4 Division of Endocrinology and Diabetology, Department of Internal Medicine, Medical University of Graz, 8036 Graz, Austria

**Keywords:** chronic heart failure, HFrEF, right ventricular function

## Abstract

**Background:** In patients with stable chronic heart failure with a reduced ejection fraction (HFrEF), left ventricular ejection fraction (LVEF) provides limited prognostic value, especially in patients with moderately to severely reduced LVEF. Echocardiographic parameters of right ventricular function may be associated with adverse clinical events in these patients. Therefore, we analyzed 164 patients with HFrEF in a prospective single-center cohort study to evaluate whether the parameters of right ventricular function are associated with worsening heart failure (WHF) hospitalizations, cardiovascular and all-cause deaths and combined endpoints. **Methods:** Echocardiographic cine loops were analyzed using vendor-independent post-processing software. Multivariate Cox regression analyses were performed, which were then adjusted for clinical characteristics and left ventricular functional parameters. **Results:** In these models, higher tricuspid annular plane systolic excursion (TAPSE) was significantly associated with lower rates of WHF hospitalizations (HR 0.880, 95%CI 0.800–0.968, *p* = 0.008), a composite endpoint of WHF hospitalizations and cardiovascular death (HR 0.878, 95%CI 0.800–0.964, *p* = 0.006), and a composite endpoint of WHF hospitalization and all-cause death (HR 0.918, 95%CI 0.853–0.988, *p* = 0.023). These associations were more pronounced in patients with LVEF ≤ 35%. **Conclusions:** In conclusion, in patients with HFrEF, TAPSE is an independent prognosticator for adverse clinical outcomes, warranting further studies to elucidate whether incorporating TAPSE into established risk scores improves their diagnostic accuracy.

## 1. Introduction

Heart failure is a major concern to public health as it is one of the leading causes of mortality and hospitalization worldwide, with an ever-increasing prevalence due to the aging of the population [1,2]. The guidelines of the European Society of Cardiology (ESC) traditionally distinguish heart failure based on left ventricular ejection fraction (LVEF) [3]. In heart failure with reduced ejection fraction (HFrEF), impairment of left ventricular systolic function and cardiac output leads to a wide range of symptoms and an overall poor prognosis [4,5,6]. Therefore, transthoracic echocardiography (TTE) plays an essential role in the assessment of those patients. Currently, LVEF guides diagnosis and therapeutic decisions in patients with heart failure [3,4]; however, at both ends of the spectrum, it lacks additional prognostic information [7,8]. Other echocardiographic parameters have proven to be more significant in predicting the clinical outcomes of patients with heart failure, such as myocardial deformation imaging and parameters of right ventricular (RV) function [7,9,10,11]. However, studies on this topic mainly report on preselected cohorts, e.g., only including patients in sinus rhythm or excluding patients with chronic kidney disease or other relevant comorbidities in heart failure [12].

The aim of the present study is to evaluate in a contemporary outpatient clinic cohort of patients with stable HFrEF whether echocardiographic parameters of RV function adjusted for standard parameters of left ventricular systolic and diastolic function are associated with the risk of mortality and hospitalization due to worsening heart failure (WHF) to address the need for data on “real-world” heart failure patients.

## 2. Materials and Methods

### 2.1. Study Population

**Figure 1:**
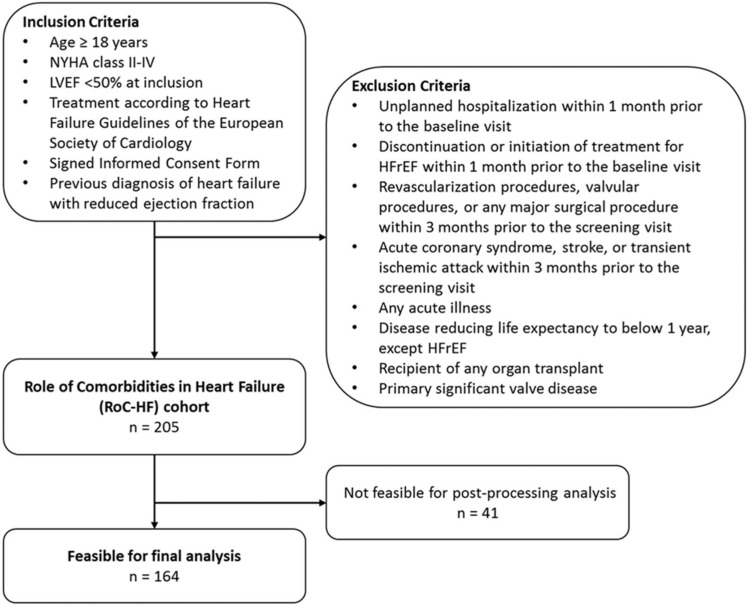
A flowchart of patient enrolment is shown.

This study is based on the Role of Comorbidities in Heart Failure (RoC-HF) study [13]. The RoC-HF study is a prospective single-center cohort study conducted at the heart failure outpatient clinic of the academic tertiary referral center of the Division of Cardiology of the Medical University of Graz. Between September 2016 and December 2018, a total of 205 consecutive patients were enrolled. The main inclusion criteria were age above 18 years, symptomatic heart failure according to New York Heart Association (NYHA) grade II–IV, an LVEF below 50% at the time of their first visit, previously diagnosed HFrEF requiring optimization of heart failure therapy, and initiated guideline-directed heart failure treatment according to the 2016 ESC Guidelines [14]. The main exclusion criteria included unplanned hospitalization and the discontinuation or initiation of a pharmacological or device treatment within one month prior to the first visit; coronary or peripheral revascularization, valvular procedures, any major surgical procedures, acute coronary syndrome, stroke, or transient ischemic attack within three months prior to the baseline visit; acute illness, recipients of an organ transplant, primary significant valve disease (moderate to severe), and diseases reducing the estimated lifespan below one year (except heart failure) (see Figure 1).

All patients provided written informed consent for participation. The permission to perform the study was granted by the Ethics Committee of the Medical University of Graz (28-467ex15/16), and written informed consent was obtained from all patients. The study was conducted in compliance with Good Clinical Practice and the Declaration of Helsinki.

### 2.2. Echocardiographic Assessment

The RoC-HF study procedures included a systematic TTE examination. The echocardiography study protocol included 2D and Doppler image acquisition in standardized transthoracic and subcostal angulations, according to current guidelines [15,16]. Post-processing analysis, including LVEF and left ventricular global longitudinal strain (LV GLS), was performed by a dedicated analyst blinded to patients’ clinical characteristics, as reported previously by our working group [17,18]. LVEF was acquired using vendor-independent post-processing software from TomTec (TOMTEC Imaging Systems, Munich, Germany). Assessment of LV GLS was performed with the vendor-independent post-processing software 2D Cardiac Performance Analysis (2DCPA) from TomTec (Version 42.00). This analysis was performed twice in each patient on different cardiac cycles of the same cine-loops, if assessable and available, and reported as mean values. LV GLS was calculated as endomyocardial GLS in a 16-segment model using the entire endomyocardial contour length, computing left ventricular deformation obtained from the apical four-, three-, and two-chamber views. Other echocardiographic parameters including heart chamber dimensions, E/e’, left atrial volume index (LAVI), peak tricuspid regurgitate velocity (TR-Vmax), systolic pulmonary artery pressure (sPAP), and tricuspid annular plane systolic excursion (TAPSE) were measured during the examination. For the present analysis, all patients in the RoC-HF study with cine-loops suitable for post-processing analysis were included.

### 2.3. Laboratory Parameters

The assessment of laboratory parameters was limited to N-terminal pro-hormone of brain natriuretic peptide (NT-proBNP), creatinine, and estimated glomerular filtration rate (eGFR) for the present analysis. Blood sampling was performed on the first study visit, and laboratory parameters were immediately determined at the Clinical Institute of Medical and Chemical Laboratory Diagnostics.

### 2.4. Follow-Up

Patient outcomes were retrieved from medical and health insurance records. Cardiovascular death was defined as a cardiovascular event (e.g., myocardial infarction, sudden cardiac death, heart failure, stroke, arrhythmias) as a primary cause of death. In patients with non-documented death, available telemedicine data of patients with implantable cardioverter-defibrillator (ICD) was interrogated postmortem. Causes of death were adjudicated by an experienced cardiologist who was blinded to the patient data (D.v.L.). If no data were available on the cause of death, death was classified as unknown. Hospitalization due to WHF was defined as an unscheduled hospitalization due to documented signs and symptoms of heart failure with at least 24 h of in-hospital stay and initiated or significantly augmented heart failure therapy [19]. The primary composite endpoint was defined as hospitalization due to WHF and cardiovascular death (composite endpoint 1). Secondary outcomes comprised a composite endpoint of hospitalization due to WHF and all-cause death (composite endpoint 2), hospitalization due to WHF, and all-cause death.

### 2.5. Statistical Analysis

All of the data are illustrated using descriptive statistics. Continuous variables are expressed as mean and standard deviation or median and interquartile range, as appropriate. Categorical variables are shown as percentages. Tests used for the normal distribution of variables included Kolmogorov–Smirnov and Shapiro–Wilk tests and visual inspections of kurtosis and skewness. Continuous variables were compared using Student’s *t* test or their non-parametric equivalents, and categorical variables were compared using the Chi-square test or Fisher’s Exact test, as appropriate.

Cox proportional hazard analyses were used to assess the associations of the echocardiographic parameters with the primary and secondary outcomes. Echocardiographic variables perceived as clinically important and significant in univariate Cox regressions were analyzed and included TAPSE, TR-Vmax, sPAP, LVEF, and LV GLS. These parameters were further individually included in a multivariate Cox regression model adjusted for age (years), gender (male/female), body mass index (kg/m^2^), atrial fibrillation, eGFR (mL/min/1.73 m^2^), and NT-proBNP (pg/mL) (model 1). RV parameters (TAPSE, TR-Vmax, and sPAP) were additionally included in a multivariate model adjusted for parameters included in model 1, and LVEF (%), LV GLS (%), E/e’, and LAVI (mL/m^2^) (model 2). 

Incidence rates for clinical events across the spectrum of TAPSE were assessed through the use of Poisson regression models with and without adjustment for age, sex, body mass index, atrial fibrillation, LVEF, eGFR, LV GLS, E/e’, and LAVI, using restricted cubic splines with 3 knots placed at the 10th, 50th, and 90th percentiles.

Statistical analyses were performed using IBM SPSS Statistics Version 27 (IBM Corporation, Armonk, NY, USA) and Stata Version 17.0 (Stata Corp., Houston, TX, USA). Results were considered statistically significant with a two-sided *p*-value < 0.05.

## 3. Results

### 3.1. Study Population

The analyzed cohort comprised a total of 164 patients. Fourty-one patients were excluded from the analysis because post-processing measurements were not feasible. The mean age of the patients was 64.8 ± 10.4 years, with a predominance of men (78%) in the sample and a mean history of heart failure of 9.0 ± 7.0 years. Ninety-eight patients (59.8%) had a non-ischemic origin of heart failure. Most patients presented with NYHA II (67%) and without angina (82%). Atrial fibrillation was present in 69 patients (42%), 112 patients (68%) presented with arterial hypertension, 78 patients (48%) had hyperlipidemia, and 44 patients (27%) presented with diabetes mellitus. All but 1 patient received at least one guideline-based heart failure drug medication, while 137 patients (84%) received a combination of at least three guideline-based heart failure drugs, consisting of either beta-blockers, mineral receptor antagonists, diuretics, and angiotensin-converting enzyme inhibitors or angiotensin receptor blockers or angiotensin neprilysin inhibitors. Six patients (4%) received sodium-glucose co-transporter 2 (SGLT2) inhibitors. Of note, those patients received SGLT2 inhibitors in the context of diabetes treatment. In this study, 111 patients (68%) had received device therapy in the form of either a pacemaker or an ICD, with or without resynchronization function. The laboratory parameters showed a median NT-proBNP (interquartile range) of 978 (332–2279) pg/mL, a mean creatinine of 1.25 ± 0.55 mg/dL, and a mean eGFR (CKD-EPI equation) of 65 ± 22 mL/min/1.73 m^2^. In the echocardiographic analysis, mean LVEF was 35.8 ± 8.2%, mean LV GLS was −12.1 ± 3.6%, mean E/e’ was 16 ± 8, mean TR-Vmax was 2.7 ± 0.5 m/sec, mean sPAP was 41 ± 12 mmHg mean LAVI was 52 ± 21 mL/m^2^, and mean TAPSE was 20 ± 5 mm, with 47 patients (29%) showing abnormal values <17 mm. Detailed baseline characteristics are displayed in Table 1.

### 3.2. Outcome Analysis

The median observation time was 4.9 (4.0–5.3) years. Forty-three patients (26%) met composite endpoint 1, sixty-three (38%) patients met composite endpoint 2, forty patients (24%) experienced hospitalization due to WHF, and forty-four patients (27%) died from any cause.

**Figure 2:**
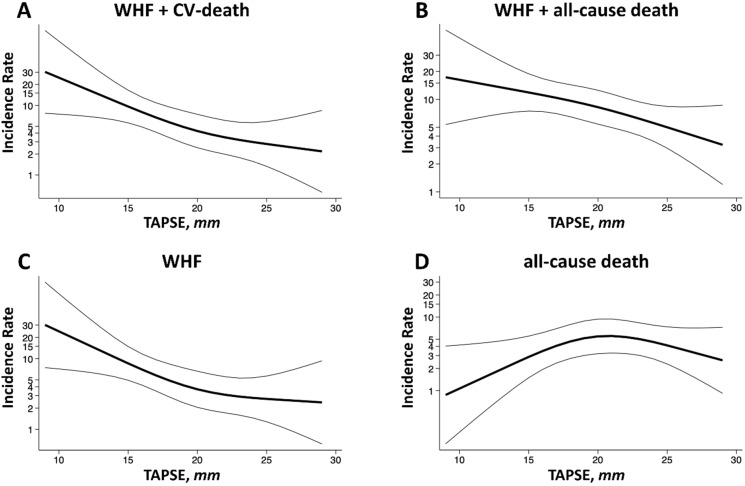
Incidence rates of clinical outcomes via tricuspid annular plane systolic excursion. Incidence rates of (**A**) composite endpoint I of worsening heart failure hospitalization or cardiovascular death, (**B**) composite endpoint II of worsening heart failure hospitalization or all-cause death, (**C**) worsening heart failure hospitalization, and (**D**) all-cause death across a range of TAPSE. Incidence rates are shown per 100 patient-years. Estimates were obtained from Poisson regression models with TAPSE expressed using restricted cubic splines. Models were adjusted for age, gender, body mass index, atrial fibrillation, eGFR, NT-proBNP, LVEF, LV GLS, E/e’, and LAVI. eGRF: estimated glomerular filtration rate; LAVI: left atrial volume index; LVEF: left ventricular ejection fraction; LV GLS: left ventricular global longitudinal strain; and TAPSE: tricuspid annular plane systolic excursion.

**Figure 3:**
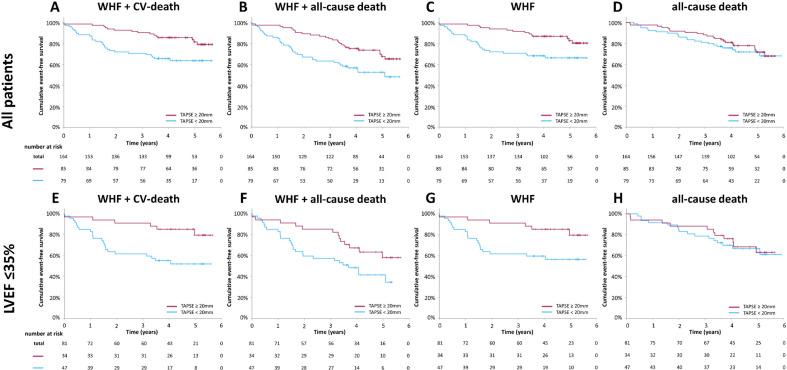
Cumulative incidence of clinical endpoints via tricuspid annular plane systolic excursion. Cumulative incidence according to TAPSE in all patients (**A**–**D**) and patients with a left ventricular ejection fraction (LVEF) ≤35% (**E**–**H**). TAPSE is stratified by mean (20 mm). TAPSE: tricuspid annular plane systolic excursion.

In univariate analyses, TAPSE, TR-Vmax, sPAP, LVEF, and LV GLS were all significantly associated with composite endpoint 1 when assessing all patients. In adjusted model 1, TAPSE (HR 0.884, 95%CI 0.817–0.958, *p* = 0.002), sPAP (HR 1.038, 95%CI 1.007–1.071, *p* = 0.017), LVEF (HR 0.959, 95%CI 0.929–0.997, *p* = 0.036), and LV GLS (HR 1.131, 95%CI 1.028–1.249, *p* = 0.011) met significance. In adjusted model 2, only TAPSE remained significant (HR 0.878, 95%CI 0.800–0.964, *p* = 0.006). TAPSE was also significantly associated with composite endpoint 2 (HR 0.918, 95%CI 0.853–0.988, *p* = 0.023) and WHF (HR 0.880, 95%CI 0.800–0.968, *p* = 0.008). In Poisson model-based analyses, lower TAPSE was associated with an increased risk of cardiovascular endpoints (Figure 2). Stratifying the cohort by means of TAPSE, those with TAPSE < 20 mm compared to those with TAPSE ≥ 20 mm had a significantly higher risk of cardiovascular endpoints, as shown in Figure 3.

Regarding all-cause death, only TR-Vmax and sPAP met significance in univariate analysis, whereas no echocardiographic parameter remained significant in adjusted model 2 (see Table 2*).*

### 3.3. Outcomes in a Subgroup with a Left Ventricular Ejection Fraction ≤ 35%

In this cohort, 81 patients (49%) showed an LVEF ≤ 35%, with a mean LVEF of 29.0 ± 4.7% in this subgroup. Those patients had a significantly longer history of heart failure (11.0 ± 7.0 versus 7.1 ± 6.4 years, *p* ≤ 0.001) and more often had a history of a previous implantation of an ICD (73 versus 57%, *p* = 0.032). They had higher NT-proBNP levels (1583 [612–3266] versus 511 [200–1507] pg/mL, *p* ≤ 0.001) and lower in-office blood pressure (systolic blood pressure 118 ± 16 versus 127 ± 23 mmHg, *p* = 0.006; diastolic blood pressure 74 ± 11 versus 79 ± 14 mmHg, *p* = 0.007). Overall, they showed significantly worse echocardiographic structural and functional parameters when compared to those with a higher LVEF, as shown in detail in Table 1.

In this subgroup, 28 patients (35%) met composite endpoint 1, 40 patients (49%) met composite endpoint 2, 26 patients (32%) underwent hospitalization due to WHF, and 27 patients (33%) died from any cause.

In patients with an LVEF ≤ 35%, TAPSE and sPAP were associated with both composite endpoints in univariate analysis, and only TAPSE was associated with WHF. LVEF and LV GLS had no additional prognostic value in this subgroup. In adjusted Cox regression models, TAPSE remained significant in composite endpoint 1 (HR 0.791, 95%CI 0.680–0.920, *p* = 0.002), composite endpoint 2 (HR 0.840, 95%CI 0.749–0.941, *p* = 0.003), and WHF (HR 0.781, 95%CI 0.667–0.916, *p* = 0.002) (Table 2).

## 4. Discussion

In this contemporary cohort of 164 patients with chronic heart failure, right ventricular systolic impairment, as defined by reduced TAPSE, showed a strong association with WHF and composite endpoints of (1) WHF and cardiovascular death and (2) WHF and all-cause mortality. This association was pronounced in patients with LVEF ≤ 35%. There was no association between TAPSE and all-cause death. The prognostic relevance of RV function in HFrEF has been documented previously, but many studies have been limited by potential selection bias, retrospective design, and lack of adjustment for left ventricular function [7,9,10,11]. The present analysis extends the existing literature and shows that TAPSE predicts cardiovascular events in an unselected cohort of patients with optimally treated chronic HFrEF. On the other hand, LVEF and LV GLS only showed poor predictive value and no prognostic value in patients with an LVEF ≤ 35% in adjusted models in this cohort.

The observed association between TAPSE and cardiovascular outcomes in chronic heart failure is in line with and extends the work of previous studies. Lundorff et al. reported RV dysfunction to be a predictor of mortality in patients with HFrEF, with TAPSE being the strongest prognostic parameter in women [9]. Other studies confirmed that RV parameters are independent predictors of poor outcomes in heart failure over the whole spectrum of the ejection fraction [10,20,21,22,23]. Furthermore, RV functional parameters may be stronger predictors of exercise capacity in HFrEF patients, which is a well-defined biomarker in the prognosis of HFrEF and in the indication of heart transplant [24,25]. A significant association between TAPSE and cardiovascular deaths in a general population without heart failure could also be established [26]. Of note, almost all reported studies measured different RV functional parameters (TAPSE, RV GLS, and the right ventricular–pulmonary artery coupling TAPSE/sPAP) and most times only highlighted those that were the best predictors. Furthermore, different cut-off values for TAPSE were used in most of those studies, and they mainly report on retrospective cohorts. Due to its prospective design and enrolment of well-characterized patients with stable chronic heart failure, our study complements the existing literature.

### 4.1. Prognostic Value of Right Ventricular Function in Chronic Heart Failure

Given that patients were included in the RoC-HF study based on their LVEF, the studied cohort includes patients over a broad and unselected spectrum of RV function. The observed association between RV function and heart failure outcomes might hypothetically reflect a causal relationship based on several mechanisms. Left ventricular failure leads to increased filling pressure, pulmonary pressure, and right ventricular afterload. Since RV function is particularly sensitive to changes in afterload, RV failure may follow left ventricular failure, even if the RV is not directly involved in the underlying left ventricular disease [27,28]. Despite the significant additional predictive value of RV function in patients with heart failure, it has not been applied in any current heart failure risk score. Established scores, such as the PARADIGM Risk of Events and Death in the Contemporary Treatment of Heart Failure (PREDICT-HF) score, Seattle Heart Failure Model (SHFM), the Cardiac and Comorbid Conditions Heart Failure (3C-HF) score, or the Meta-Analysis Global Group in Chronic Heart Failure (MAGGIC-HF) score for risk-stratification in patients with heart failure rely only on LVEF [29,30,31,32]. Whether adding RV function improves the diagnostic accuracy of heart failure scores needs to be proven in future studies. TAPSE could qualify as a valid additional parameter in these risk scores, as its assessment is non-invasive, reliable, simple, and can be achieved via far less technician- and insonation-angle-dependent methods in comparison to other parameters and indices such as RV myocardial deformation imaging.

### 4.2. Normal Right Ventricular Systolic Function in Heart Failure

The normal value of RV systolic function assessed using TAPSE in a general population is defined as 24 ± 3.5 mm, while TAPSE < 17 mm indicates reduced RV systolic function [15]. In this cohort, incidence rates increased with a TAPSE < 20 mm, which is in the supposedly normal range. There are no large cohort studies on a normal TAPSE range in patients with heart failure. Whether normal ranges for RV function should be redefined in the context of coexisting HFrEF should be investigated in future studies. Patients with heart failure, especially with HFrEF, are at higher risk of cardiovascular events compared to a general, healthy population. Therefore, in those patients, a different cut-off may be beneficial as moderately impaired RV systolic function may point to worse outcomes. This should be further considered and verified in large cohort studies with a prospective study design, which may lead to more intensified monitoring or follow-ups to avoid hospitalizations due to heart failure.

### 4.3. Strengths and Limitations

This cohort is a contemporary outpatient clinic heart failure cohort that was only preselected based on LVEF screening to give valid insight into “real world” heart failure patient outcomes; however, due to optimal heart failure treatment, mortality in this cohort was lower overall when compared to results in the literature [33,34,35]. Another limitation is related to the timing of the inclusion period used in this study; practically no patient in this cohort received SGLT2 inhibitors as heart failure therapy at the time point of the TTE since SGLT2 inhibitors were introduced into routine clinical care for heart failure patients afterward. 

In contrast to previous investigations, our study did not show a significant association between TAPSE and all-cause mortality, but only with cardiovascular events. This may be explained by the low number of deaths in this cohort.

Our data are limited as we did not assess right ventricular function using new techniques, such as 3D-EXO or speckle-tracking echocardiography. This should be assessed in future studies on this topic.

## 5. Conclusions

Despite the fact that HFrEF is considered to be a primarily left ventricular pathology, echocardiographic parameters of the left ventricle provide poor additional prognostic value in terms of clinical outcomes. RV systolic function is a particularly valuable prognostic parameter in patients with worse LVEF, indicating that as ventricular function declines, it is equally important to consider RV functional parameters in the prediction of cardiovascular events. In this study, TAPSE was the strongest independent prognostic echocardiographic parameter for clinical outcomes in patients with heart failure, particularly among patients with an LVEF lower than 35%. Therefore, TAPSE could serve as an additional prognostic marker, warranting further studies to elucidate whether incorporating TAPES into established risk scores improves their diagnostic accuracy.

## Figures and Tables

**Table 1:** Baseline characteristics.

	All Patients	LVEF > 35%	LVEF ≤ 35%	*p*-Value *
	*n* = 164	*n* = 83	*n* = 81	
**Demographics**				
Female, *n* (%)	36 (22)	23 (28)	13 (16)	0.090
Age, years	64.8 ± 10.4	64.8 ± 10.7	64.8 ± 10.1	0.927
BMI, kg/m^2^	28.5 ± 4.7	28.8 ± 5.1	28.2 ± 4.3	0.638
Heart failure duration, years	9.0 ± 7.0	7.1 ± 6.4	11.0 ± 7.0	**<0.001**
Caucasian ethnicity, *n* (%)	164 (100)	83 (100)	81 (100)	**-**
**Symptoms**				
NYHA functional class				
NYHA II, *n* (%)	110 (67)	57 (69)	53 (65)	0.563
NYHA II-III, *n* (%)	30 (18)	13 (16)	17 (21)	
NYHA III, *n* (%)	23 (14)	13 (16)	10 (12)	
NYHA IV, *n* (%)	1 (1)	0 (0)	1 (1)	
Angina, *n* (%)	30 (18)	16 (20)	14 (17)	0.498
**Clinical characteristics**				
Ischemic-origin, *n* (%)	66 (40)	30 (36)	36 (44)	0.279
Non-ischemic-origin, *n* (%)	98 (60)	53 (64)	45 (56)	
SBP, mmHg	123 ± 20	127 ± 23	118 ± 16	**0.006**
DBP, mmHg	77 ± 13	79 ± 14	74 ± 11	**0.007**
24 h SBP, mmHg	111 ± 13	114 ± 14	108 ± 12	**0.003**
24 h DBP, mmHg	68 ± 9	69 ± 9	67 ± 9	0.066
Heart rate, bpm	66 ± 12	64 ± 11	67 ± 13	0.194
24 h-heart rate, bpm	67 ± 10	68 ± 11	66 ± 9	0.349
**Device therapy**				
Pacemaker, *n* (%)	47 (29)	21 (25)	26 (32)	0.370
ICD, *n* (%)	106 (65)	47 (57)	59 (73)	**0.032**
CRT, *n* (%)	53 (32)	27 (33)	26 (32)	0.933
**Comorbidities**				
Atrial fibrillation, *n* (%)	69 (42)	34 (41)	35 (43)	0.874
Diabetes mellitus, *n* (%)	44 (27)	20 (24)	24 (30)	0.381
Arterial hypertension, *n* (%)	112 (68)	55 (66)	57 (70)	0.662
Hyperlipidemia, *n* (%)	78 (48)	44 (53)	34 (42)	0.271
COPD, *n* (%)	34 (21)	13 (16)	21 (26)	0.253
Smoker, *n* (%)	105 (64)	56 (68)	49 (61)	0.648
**Pharmacological treatment**				
ACE/ARB/ARNI, *n* (%)	151 (92)	77 (93)	74 (91)	0.780
Beta-blocker, *n* (%)	158 (96)	81 (98)	77 (95)	0.440
MRA, *n* (%)	129 (79)	64 (77)	65 (80)	0.704
Thiazide, *n* (%)	18 (11)	8 (10)	10 (12)	0.625
Loop-diuretics, *n* (%)	96 (59)	48 (58)	48 (59)	0.875
SGLT2 inhibitors, *n* (%)	6 (4)	5 (6)	1 (1)	0.210
**Laboratory parameters**				
NT-proBNP, pg/mL	978 (332–2279)	511 (200–1507)	1583 (612–3266)	**<0.001**
Creatinine, mg/dL	1.25 ± 0.55	1.22 ± 0.43	1.28 ± 0.65	0.669
eGFR, mL/min/1.73 m^2^	65 ± 22	64 ± 22	66 ± 23	0.644
**Echocardiography**				
LVEF, %	35.8 ± 8.2	42.5 ± 4.6	29.0 ± 4.7	**<0.001**
LVEDV, mL	155 ± 61	126 ± 38	184 ± 67	**<0.001**
LVESV, mL	102 ± 50	73 ± 24	132 ± 53	**<0.001**
GLS, %	−12.1 ± 3.6	−14.4 ± 3.0	−9.7 ± 2.6	**<0.001**
E/e’	16 ± 8	13 ± 6	18 ± 9	**<0.001**
TAPSE, mm	20 ± 5	21 ± 5	18 ± 5	**0.002**
TR-Vmax, m/s	2.7 ± 0.5	2.6 ± 0.4	2.8 ± 0.5	**0.023**
sPAP, mmHg	41 ± 12	39 ± 10	43 ± 13	**0.038**
LAVI, mL/m^2^	52 ± 21	47 ± 19	57 ± 21	**0.001**

Parameters reported in mean ± standard deviation, median (interquartile range), or frequency (percentage). ACE: angiotensin-converting enzyme inhibitor; ARB: angiotensin receptor blockers; ARNI: angiotensin neprilysin inhibitor; BMI: body mass index; COPD: chronic obstructive pulmonary disease; CRT: cardiac resynchronization therapy; DBP: diastolic blood pressure; eGFR: estimated glomerular filtration rate; GLS: global longitudinal strain; ICD: implantable cardioverter defibrillator; LAVI: left atrial volume index; LVEDV: left ventricular end-diastolic volume; LVEF: left ventricular ejection fraction; LVESV: left ventricular end-systolic volume; MRA: mineral receptor antagonist; NT-proBNP: N-terminal pro-brain natriuretic peptide; NYHA: New York Heart Association; SBP: systolic blood pressure; SGLT2: sodium-glucose co-transporter 2 inhibitor; sPAP: systolic pulmonary artery pressure; and TAPSE: tricuspid annular plane systolic excursion; TR-Vmax: maximal tricuspid regurgitation velocity. * Student’s-*t*-test; Chi-square test.

**Table 2:** Univariate and adjusted Cox regression models.

	All Patients			LVEF ≤ 35%		
	Univariate	Model 1 *	Model 2 **	Univariate	Model 1 *	Model 2 **
	Hazard Ratio (95% Confidence Interval), *p*-Value			Hazard Ratio (95% Confidence Interval), *p*-Value		
**Composite endpoint I**						
TAPSE	0.859 (0.800–0.921) *p* ≤ 0.001	0.884 (0.817–0.958) *p* = 0.002	0.878 (0.800–0.964), *p* = 0.006	0.838 (0.761–0.923), *p* ≤ 0.001	0.836 (0.749–0.933), *p* = 0.001	0.791 (0.680–0.920), *p* = 0.002
TR-Vmax	2.168 (1.082–4.348) *p* = 0.029	1.737 (0.781–3.863) *p* = 0.176	1.375 (0.526–3.593), *p* = 0.516	2.246 (0.947–5.327), *p* = 0.066		
sPAP	1.043 (1.017–1.069) *p* ≤ 0.001	1.038 (1.007–1.071) *p* = 0.017	1.032 (0.996–1.070), *p* = 0.082	1.037 (1.008–1.068), *p* = 0.013	1.058 (1.016–1.101), *p* = 0.006	1.059 (1.006–1.115), *p* = 0.028
LVEF	0.953 (0.920–0.987) *p* = 0.008	0.959 (0.929–0.997) *p* = 0.036		0.982 (0.912–1.058), *p* = 0.633		
LV GLS	1.152 (1.058–1.255) *p* = 0.001	1.131 (1.028–1.249) *p* = 0.011		1.068 (0.927–1.231), *p* = 0.361		
**Composite endpoint II**						
TAPSE	0.885 (0.836–0.937) *p* ≤ 0.001	0.902 (0.844–0.964) *p* = 0.002	0.918 (0.853–0.988), *p* = 0.023	0.890 (0.825–0.960), *p* = 0.003	0.894 (0.823–0.972), *p* = 0.008	0.840 (0.749–0.941), *p* = 0.003
TR-Vmax	2.445 (1.406–4.250) *p* = 0.002	2.199 (1.133–4.265), *p* = 0.020	1.903 (0.820–4.418), *p* = 0.134	2.060 (1.053–4.029), *p* = 0.035	2.498 (1.006–6.202), *p* = 0.048	3.080 (1.064–8.915), *p* = 0.038
sPAP	1.039 (1.019–1.060) *p* ≤ 0.001	1.033 (1.007–1.060), *p* = 0.013	1.025 (0.994–1.057), *p* = 0.113	1.025 (1.002–1.050), *p* = 0.037	1.035 (1.001–1.070), *p* = 0.046	1.037 (0.998–1.078), *p* = 0.066
LVEF	0.960 (0.932–0.988) *p* = 0.005	0.974 (0.942–1.006), *p* = 0.112		1.016 (0.948–1.088), *p* = 0.654		
LV GLS	1.106 (1.033–1.184) *p* = 0.004	1.071 (0.989–1.160), *p* = 0.092		0.989 (0.878–1.115), *p* = 0.862		
**Worsening heart failure hospitalization**						
TAPSE	0.857 (0.796–0.922) *p* ≤ 0.001	0.881 (0.811–0.957), *p* = 0.003	0.880 (0.800–0.968), *p* = 0.008	0.836 (0.756–0.925), *p* ≤ 0.001	0.830 (0.739–0.931), *p* = 0.002	0.781 (0.667–0.916), *p* = 0.002
TR-Vmax	2.015 (0.984–4.125) *p* = 0.055			1.737 (0.712–4.236), *p* = 0.225		
sPAP	1.038 (1.012–1.065) *p* = 0.004	1.030 (0.999–1.063), *p* = 0.059	1.030 (0.992–1.069), *p* = 0.121	1.027 (0.995–1.059), *p* = 0.095		
LVEF	0.951 (0.917–0.987) *p* = 0.007	0.958 (0.920–0.998), *p* = 0.038		0.983 (0.910–1.061), *p* = 0.659		
LV GLS	1.165 (1.066–1.273) *p* ≤ 0.001	1.146 (1.037–1.265), *p* = 0.007		1.074 (0.927–1.244), *p* = 0.342		
**All-cause mortality**						
TAPSE	0.954 (0.896–1.016) *p* = 0.140			0.959 (0.882–1.042), *p* = 0.321		
TR-Vmax	2.226 (1.155–4.288) *p* = 0.017	2.817 (1.209–6.559), *p* = 0.016	2.735 (0.942–7.942), *p* = 0.064	1.486 (0.674–3.278), *p* = 0.326		
sPAP	1.029 (1.005–1.053) *p* = 0.016	1.030 (0.999–1.062), *p* = 0.059	1.025 (0.986–1.066), *p* = 0.210	1.009 (0.981–1.038), *p* = 0.528		
LVEF	0.979 (0.945–1.014) *p* = 0.226			1.053 (0.957–1.158), *p* = 0.289		
LV GLS	1.045 (0.964–1.132) *p* = 0.283			0.957 (0.827–1.107), *p* = 0.555		

Univariate Cox regression analysis and adjusted models for composite endpoint I (worsening heart failure hospitalization or cardiovascular death), composite endpoint II (worsening heart failure hospitalization or all-cause death), worsening heart failure hospitalization, and all-cause mortality. BMI: body mass index; eGFR: estimated glomerular filtration rate; LV GLS: left ventricular global longitudinal strain; LAVI: left atrial volume index; LVEF: left ventricular ejection fraction; NT-proBNP: N-terminal pro-brain natriuretic peptide; sPAP: systolic pulmonary artery pressure; TAPSE: tricuspid annular plane systolic excursion; TR-Vmax: maximal tricuspid regurgitation velocity. * adjusted for age, gender, BMI, atrial fibrillation, eGFR, and NT-proBNP. ** adjusted for age, gender, BMI, atrial fibrillation, GFR, NT-proBNP, LVEF, LV GLS, E/e’, and LAVI.

## Data Availability

The data underlying this article will be shared upon reasonable request to the corresponding author.
